# Molecular insights in the pathogenesis of classical Ehlers-Danlos syndrome from transcriptome-wide expression profiling of patients’ skin fibroblasts

**DOI:** 10.1371/journal.pone.0211647

**Published:** 2019-02-04

**Authors:** Nicola Chiarelli, Giulia Carini, Nicoletta Zoppi, Marco Ritelli, Marina Colombi

**Affiliations:** Department of Molecular and Translational Medicine, Division of Biology and Genetics, University of Brescia, Brescia, Italy; Northwestern University, UNITED STATES

## Abstract

Classical Ehlers-Danlos syndrome (cEDS) is a dominant inherited connective tissue disorder mainly caused by mutations in the *COL5A1* and *COL5A2* genes encoding type V collagen (COLLV), which is a fibrillar COLL widely distributed in a variety of connective tissues. cEDS patients suffer from skin hyperextensibility, abnormal wound healing/atrophic scars, and joint hypermobility. Most of the causative variants result in a non-functional *COL5A1* allele and COLLV haploinsufficiency, whilst *COL5A2* mutations affect its structural integrity. To shed light into disease mechanisms involved in cEDS, we performed gene expression profiling in skin fibroblasts from four patients harboring haploinsufficient and structural mutations in both disease genes. Transcriptome profiling revealed significant changes in the expression levels of different extracellular matrix (ECM)-related genes, such as *SPP1*, *POSTN*, *EDIL3*, *IGFBP2*, and *C3*, which encode both matricellular and soluble proteins that are mainly involved in cell proliferation and migration, and cutaneous wound healing. These gene expression changes are consistent with our previous protein findings on *in vitro* fibroblasts from other cEDS patients, which exhibited reduced migration and poor wound repair owing to COLLV disorganization, altered deposition of fibronectin into ECM, and an abnormal integrin pattern. Microarray analysis also indicated the decreased expression of *DNAJB7*, *VIPAS39*, *CCPG1*, *ATG10*, *SVIP*, which encode molecular chaperones facilitating protein folding, enzymes regulating post-Golgi COLLs processing, and proteins acting as cargo receptors required for endoplasmic reticulum (ER) proteostasis and implicated in the autophagy process. Patients’ cells also showed altered mRNA levels of many cell cycle regulating genes including *CCNE2*, *KIF4A*, *MKI67*, *DTL*, and *DDIAS*. Protein studies showed that aberrant COLLV expression causes the disassembly of itself and many structural ECM constituents including COLLI, COLLIII, fibronectin, and fibrillins. Our findings provide the first molecular evidence of significant gene expression changes in cEDS skin fibroblasts highlighting that defective ECM remodeling, ER homeostasis and autophagy might play a role in the pathogenesis of this connective tissue disorder.

## Introduction

Classical Ehlers-Danlos syndrome (cEDS) (MIM #130000) is an autosomal dominant connective tissue disorder with an estimated prevalence of 1:20,000 and is mainly characterized by abnormal skin texture and joint hypermobility (JHM). According to the 2017 EDS nosology [1], skin hyperextensibility plus atrophic scarring (criterion 1) and generalized JHM (criterion 2) are major criteria for cEDS, whereas easy bruising, soft and doughy skin, skin fragility, molluscoid pseudotumors, subcutaneous spheroids, hernia, epicanthal folds, and JHM complications, such as sprains, luxation/subluxation, pain, and family history of a first-degree relative are minor criteria. Minimal criteria suggestive for cEDS are major criterion 1 plus either major criterion 2 and/or at least three minor criteria. Confirmatory molecular testing is mandatory to reach a final diagnosis [1–3].

More than 90% of cEDS patients harbor a pathogenic variant in the *COL5A1* and *COL5A2* genes encoding type V collagen (COLLV) [4,5]. Besides, in a minority of cEDS patients, the specific *COL1A1* c.934C>T (p.Arg312Cys) variant is found [1,5,6]. COLLV haploinsufficiency is the most common molecular defect caused by a non-functional *COL5A1* allele, whereas variants in *COL5A2* affect its structural integrity and are generally associated with a more severe phenotype [4,5,7,8]. The majority of pathogenetic variants in *COL5A1* are nonsense, frameshift or splice site mutations that generate a premature termination codon, with the consequent activation of the nonsense-mediated mRNA decay pathway, finally leading to COLLV haploinsufficiency [2,4,5]. Conversely, almost all described disease-causing variants in *COL5A2* are missense or in-frame exon-skipping splice mutations, which result in the production of mutant α2(V) chains that exert a dominant negative effect on COLLV molecules [4,5].

COLLV, classified as a regulatory fibril-forming COLL, is a heterotrimer composed of two pro-α1(V) chains and a single pro-α2(V) chain [2]. Although COLLV is a quantitatively minor fibrillar COLL, it is widely distributed in a variety of connective tissues including dermis, tendons, and muscles among the most affected tissues in cEDS patients [9]. COLLV plays a central role in fibrillogenesis forming heterotypic fibrils with other fibril-forming COLLs, particularly COLLI, since it is involved in the fibril assembly and regulation of fibril diameter [10]. COLLV knockout mice synthesize and secrete normal amounts of COLLI, but COLL fibrils are virtually absent, and mice die at the onset of organogenesis, indicating that proper fibrillogenesis regulated by COLLV is crucial for normal embryonic development [9]. The COLLV-deficient dermis has a disruption in COLL fibrillogenesis with fewer fibrils, abnormal fibril structure, *i*.*e*., “cauliflower”-shaped fibrils with an abnormal diameter distribution, and abnormal packing [11].

COLLV has also been implicated in wound healing process given its upregulated expression during tissue healing [12,13], although the exact mechanism is not yet elucidated. In this regard, we previously demonstrated that cEDS patients’ dermal fibroblasts have a reduced migration capability and delayed wound repair in a scratch assay [14] caused by poor COLLV organization, defective fibrillar fibronectin (FN) assembly into the extracellular matrix (ECM) and abnormal integrin pattern [15,16]. Likewise, both *Col5a1*- and *Col5a2*-deficient mice showed deficits in wound healing [17,18].

To shed light on altered gene expression pattern and dysregulated biological processes involved in the molecular pathology of cEDS, we carried out transcriptome profiling on dermal fibroblasts from four cEDS patients with either haploinsufficient or structural mutations in both causative genes.

## Patients, materials and methods

### Clinical evaluation of cEDS patients

Approval of the present research was obtained by the local Ethical Committee “Comitato Etico di Brescia, ASST degli Spedali Civili, Brescia, Italia”, registration number NP2658 and the study was conducted in accordance with the principles of the Declaration of Helsinki. cEDS patients (2 females, 2 males) and healthy individuals (5 females, 4 males) were evaluated in the Centre of Heritable Connective tissue disorders and Ehlers-Danlos syndromes of the Spedali Civili of Brescia. Mean age at examination was 40 years for the patients (range 34–46) and 42 years for the healthy subjects (range 32–49). Written informed consent to the study and for skin biopsy was obtained from all patients and healthy donors according to Italian bioethics laws. All of the patients fulfilled the cEDS criteria of the Villefranche nosology [19] and its 2017 revision [1], whereas healthy individuals did not show any cEDS sign. A summary of the patients’ clinical features according to the 2017 cEDS nosology is reported in **S1 Table**.

The cEDS fibroblast strains here analyzed (cEDS P1, P2, P3, P4) were obtained from previously clinically characterized patients in Ritelli et al., [4] as AN_002514, AN_002503, and AN_002526, harboring the c.2988del (p.Gly997Alafs*77), c.1165-2A>G (p.Pro389Leufs*168), and c.4178G>A (p.Gly1393Asp) *COL5A1* pathogenic variants, respectively, and as AN_002534 carrying the c.2499+2T>C (p.Gly816_Pro833del) *COL5A2* splice variant. Specifically, the c.2988del frameshift mutation and the c.1165-2A>G splice variant are null alleles leading to COLLV haploinsufficiency, whereas the c.4178G>A missense mutation and the c.2499+2T>C splice site variant are structural mutations that exert a dominant negative effect on the protein by interfering with the triple helix formation [4] and **S1 Table**.

### Cell cultures and antibodies

Skin fibroblast cultures from four cEDS patients and nine unrelated age-matched healthy donors were established in our lab from skin biopsies by standard protocols. Fibroblasts were grown *in vitro* at 37° C in a 5% CO_2_ atmosphere in Earle’s Modified Eagle Medium (MEM) supplemented with 2 mM L-glutamine, 10% FBS, 100 μg/ml penicillin and streptomycin (Life Technologies, Carlsbad, CA, USA). Cells were expanded until full confluency and then harvested by 0.25% trypsin/0.02% EDTA treatment at the same passage number (from 2^nd^ to 6^th^), as previously described [14–16].

Goat anti-COLLI, anti-COLLIII, and anti-COLLV polyclonal antibodies (Abs) were from Millipore-Chemicon Int. (Billerica, MA). Mouse anti-fibrillins (FBNs) (clone 11C1.3) monoclonal Ab (mAb) was from NeoMarkers (Fremont, CA). The rabbit Ab against human FN was from Sigma Chemicals (St. Louis, MO). Rhodamine-conjugated anti-goat IgG Ab was from Calbiochem–Novabiochem INTL, and the Alexa Fluor 488 anti-rabbit and Alexa Fluor 594 anti-mouse secondary Abs were from Life Technologies.

### Immunofluorescence microscopy (IF)

To analyze the COLLI-, COLLIII-, COLLV-, FN- and FBNs-ECM organization, cEDS fibroblasts were immunoreacted 48 h after seeding as described previously [14, 20–23]. In brief, cold methanol fixed fibroblasts were immunoreacted with 1:100 anti-FN, anti-COLLI, COLLIII, and anti-COLLV Abs. In brief, cold methanol fixed fibroblasts were immunoreacted for 1 h with 1:100 anti-COLLI, anti-COLLIII, anti-COLLV, anti-FN Abs, and with 1 μg/ml anti-FBNs mAb, which recognizes all FBN isoforms. After washing in PBS, cells were incubated for 1 h with rhodamine-conjugated anti-goat IgG or anti-rabbit or anti-mouse secondary Abs conjugated to Alexa Fluor 488 and 594, respectively. IF signals were acquired by a CCD black-and-white TV camera (SensiCam-PCO Computer Optics GmbH, Germany) mounted on a Zeiss fluorescence Axiovert microscope and digitalized by Image Pro Plus software (Media Cybernetics, Silver Spring, MD, USA). All experiments were repeated three times.

### Microarray procedures

Total RNA was purified from skin fibroblasts of four cEDS patients and nine healthy individuals using the Qiagen RNeasy kit, by standard protocol (Qiagen, Hilden, Germany). Prior to microarray analysis, quality control of RNA was checked on the Agilent 2100 BioAnalyzer (Agilent Technologies, Santa Clara, CA, USA). Transcriptome analysis was carried out by using the Affymetrix GeneChip Human Gene 1.0 ST array as previously described in detail [21–24]. The resulting CEL files were analyzed using the Partek Genomics Suite software (Partek Inc., St. Louis, MO, USA), as previously described [23]. To detect differentially expressed genes (DEGs) between cEDS *vs* control cells, a one-way ANOVA analysis was applied as a criterion for the selection of DEGs. To assess significant differences in the gene expression profile in cEDS fibroblasts, we selected genes that had more than a 1.5-fold change and p-value <0.05. Multiple testing correction was applied to control the false-discovery rate (FDR) using the Benjamini–Hochberg (BH) procedure [25]. Genes with a FDR <0.05 were selected as differentially expressed and retained for further analysis. Database for Annotation, Visualization and Integrated Discovery (DAVID) v.6.8 and ToppGene suite database were queried for identification of significantly enriched functional annotations, Gene Ontology (GO) biological process, and pathways analysis. Specifically, the main GO categories were examined with a FDR <30% after BH correction. All microarray data were MIAME compliant, and raw array data and processed data were deposited to the Gene Ontology Omnibus Database (GEO Accession Number GSE117680).

### Quantitative real-time PCR

Relative expression levels of a series of selected DEGs identified by array analysis were confirmed by quantitative real-time PCR (qPCR) using different RNA extractions obtained from skin fibroblast cultures of all patients and 6 out of 9 healthy subjects. 3 μg of total RNA were reverse-transcribed with random primers by standard procedure. qPCR was performed with SYBR Green qPCR Master Mix (Life Technologies), 10 ng of cDNA, and with 10 μM of each primer set. qPCR was performed using the ABI PRISM 7500 Real-Time PCR System by standard thermal cycling conditions: initial denaturation for 30 s at 95° C, followed by 40 cycles of 95° C for 15 s and 60° C for 60 s. *HPRT*, *GAPDH*, *ATP5B*, and *CYC1* reference genes were amplified for normalization of cDNA loading. Relative mRNA expression levels were normalized to the geometric mean of these reference genes and analyzed using the 2^-(ΔΔCt)^ method. Results were expressed as the mean value of relative quantification ± SEM. Statistical significance between groups was determined using unpaired Student’s t-test with GraphPad Prism software.

## Results

### Gene expression profiling

**Fig 1:**
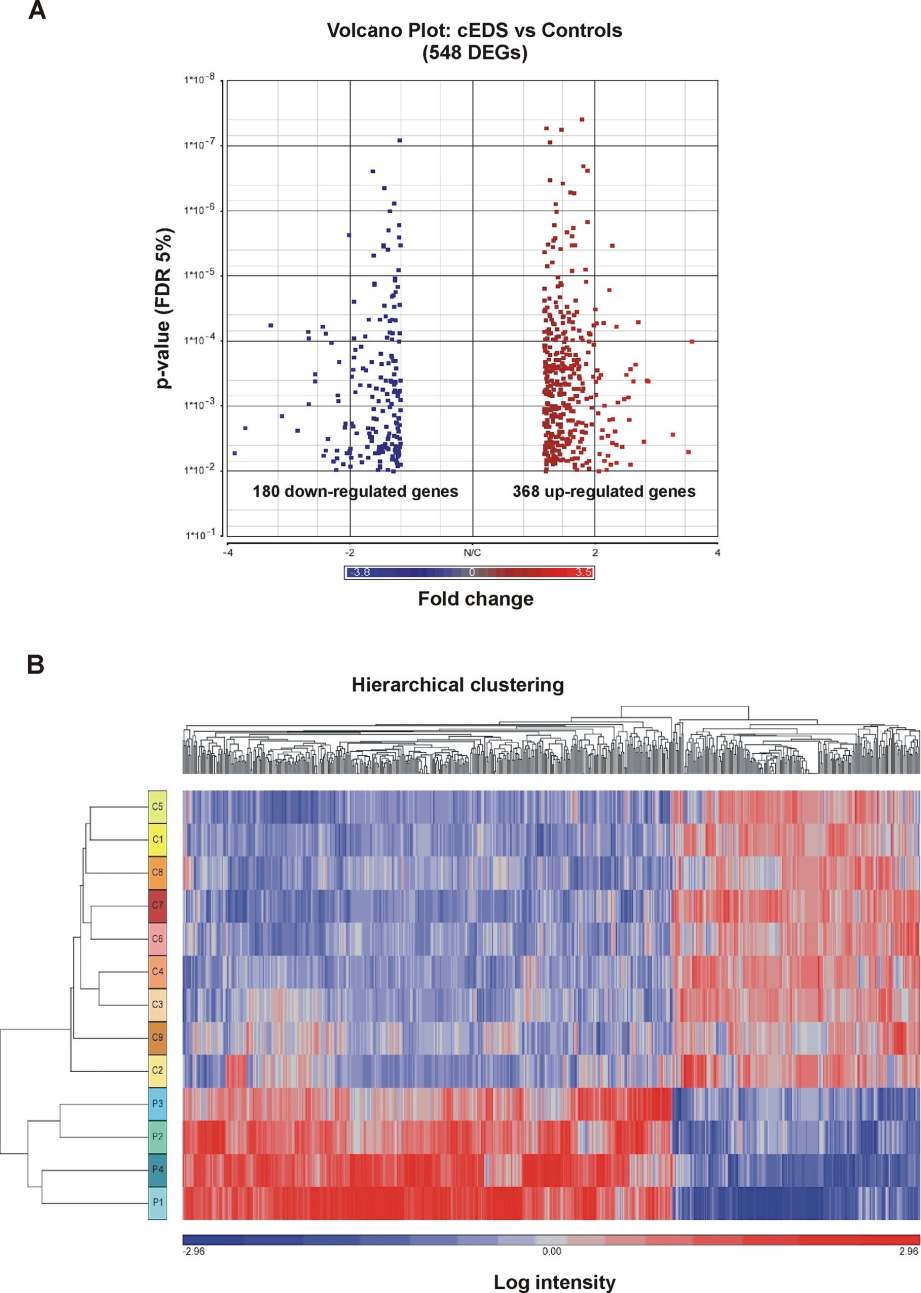
Volcano plot and hierarchical clustering analyses for DEGs in skin fibroblasts from cEDS patients and control subjects. **(A)** The volcano plot depicts all statistically significant 548 DEGs (180 down-regulated, 368 upregulated) identified in cEDS cells. The fold-change of DEGs on the x-axis *vs* the statistical significance (FDR-adjusted p-value <0.05) on the y-axis is shown; the up-regulated genes are reported in red, and the down-regulated genes are in blue. **(B)** Hierarchical clustering analysis of DEGs identified in cEDS fibroblasts. Although fibroblasts from only 9 control subjects (C) and 4 cEDS patients (P) were analyzed, this analysis showed the presence of two distinct clusters of transcripts that clearly distinguish the patients from the healthy individuals. The red color represents high gene expression, and blue represents low gene expression.

To identify genes potentially involved in the molecular mechanisms associated with the pathogenesis of cEDS, transcriptome-wide expression analysis was carried out comparing gene expression pattern among patients’ and controls’ skin fibroblasts. Gene expression profiling identified 548 DEGs in cEDS patients’ cells when compared to controls: of these, 368 genes were up-regulated, and 180 were down-regulated (**S2 Table**). **Fig 1A** represents the volcano plot that illustrates the statistical significance (FDR) *vs* fold-change of DEGs, and **Table 1** reports a selection of up- and down-regulated DEGs (FDR < 0.05 and fold change threshold ≥ 1.5). To group transcripts with similar expression profiles between patients and controls, hierarchical clustering of DEGs was conducted (**Fig 1B**).

**Table 1:** Selection of DEGs identified in cEDS patients’ skin fibroblasts.

Down-regulated genes			
Gene symbol	Gene description	FDR <0.05	Fold change
*EDIL3*	EGF-like repeats and discoidin I-like domains 3	0,005	-3,83
*RNU5B-1*	RNA, U5B small nuclear 1	0,002	-3,61
*SNORD80*	small nucleolar RNA, C/D box 80	5,80E^-05^	-3,13
*C11orf87*	chromosome 11 open reading frame 87	0,001	-2,94
*TRIB3*	Tribbles pseudokinase 3	9,08E^-05^	-2,52
*SNORD116-6*	Small nucleolar RNA, C/D box 116–6	0,002	-2,69
*GDF15*	Growth differentiation factor 15	0,0003	-2,43
*WDR74*	WD repeat domain 74	0,00007	-2,53
*WNT5A*	Wingless-type MMTV integration site family, member 5A	0,004	-2,29
*HEY1*	Hes-related family bHLH transcription factor with YRPW motif 1	0,0006	-2,14
*CCPG1*	Cell cycle progression 1	0,0001	-1,95
*TBC1D19*	TBC1 domain family, member 19	2,34E^-06^	-2,01
*HAND2*	Heart and neural crest derivatives expressed 2	0,0008	-2,13
*CSRP2*	Cysteine and glycine-rich protein 2	9,00E^-05^	-1,95
*KRT6B*	Keratin 6B, type II	2,52E^-05^	-1,95
*PAPPA*	Pregnancy-associated plasma protein A, pappalysin 1	0,001	-1,74
*ATG10*	Autophagy related 10	4,42E-^07^	-1,64
*POSTN*	Periostin, osteoblast specific factor	0,002	-1,60
*SVIP*	Small VCP/p97-interacting protein	9,51E-^05^	-1,57
*RAB33A*	RAB33A, member RAS oncogene family	0,001	-1,55
*VPS29*	VPS29 retromer complex component	0,0004	-1,54
*SPP1*	Secreted phosphoprotein 1	0,008	-1,52
*VIPAS39*	VPS33B interacting protein	3,35E-^06^	-1,50
**Up-regulated genes**			
*IGFBP2*	Insulin like growth factor binding protein 2	0,0001	3,46
*GRPR*	Gastrin-releasing peptide receptor	0,005	3,39
*C3*	Complement component 3	0,002	3,10
*CDC6*	Cell division cycle 6	0,0004	2,70
*DTL*	Denticleless E3 ubiquitin protein ligase homolog (Drosophila)	0,0004	2,67
*PODXL*	PODXL podocalyxin-like	0,003	2,63
*BRIP1*	BRCA1 interacting protein C-terminal helicase 1	5,16E^-05^	2,55
*ADAMTSL1*	ADAMTS like 1	0,0002	2,50
*CCNE2*	Cyclin E2	0,0004	2,48
*ND6*	NADH dehydrogenase, subunit 6 (complex I)	0,0002	2,44
*ZNF730*	Zinc finger protein 730	0,001	2,42
*PEG10*	Paternally expressed 10	0,0007	2,41
*GABBR2*	Gamma-aminobutyric acid (GABA) B receptor, 2	0,0003	2,38
*FAM111B*	Family with sequence similarity 111, member B	0,0007	2,36
*ESCO2*	Establishment of sister chromatid cohesion N-acetyltransferase	0,0008	2,27
*HIST1H2BM*	Histone cluster 1, H2bm	6,05E^-05^	2,26
*DCLK1*	Doublecortin-like kinase 1	0,003	2,25
*RGS4*	Regulator of G-protein signaling 4	0,007	2,25
*CCBE1*	Collagen and calcium binding EGF domains 1	0,002	2,23
*RASD2*	RASD family, member 2	0,001	2,20
*HSD17B6*	Hydroxysteroid (17-beta) dehydrogenase 6	0,003	2,20
*FAM106A*	Family with sequence similarity 106, member A	0,008	2,18
*EPHA5*	EPH receptor A5	0,002	2,15
*PTPN3*	Protein tyrosine phosphatase, non-receptor type 3	0,005	2,15
MKI67	Marker of proliferation Ki-67	0,0009	2,10

To identify biological processes that are over- or under-represented in patients’ cells, we classified up- and down-regulated genes according to the GO categories by using the DAVID and ToppGene biological databases. Only GO categories with FDR <0.3 in each annotation term were considered relevant.

Functional analysis of the 368 up-regulated genes yielded 11 different GO clusters (**Table 2** and **S3 Table**). The most prominent group of up-regulated DEGs was implicated in DNA replication, cell cycle regulation, and DNA damage and repair responses, including different cell cycle protein-encoding genes. Specifically, different genes, *i*.*e*., *CCNE2*, *CDC6*, *TYMS*, *CDC45*, *MCM8*, *POLE2*, *RRM2*, *MCM10*, *CDCA5*, *MCM4*, *MCM5*, *MCM6*, are involved in the transition of G1/S phase of cell cycle. Many genes belong to the minichromosome maintenance complex (MCM, *i*.*e*., *MCM8*, *MCM4*, *MCM5*, *MCM6*), which is a key component of the pre-replication complex required for the initiation of eukaryotic DNA replication. We also found that GO categories related to “DNA synthesis involved in DNA repair” and “double-strand break repair via homologous recombination” were significantly enriched with different up-regulated genes (*EXO1*, *RFC3*, *XRCC2*, *RAD51AP1*, *BLM*, *BRIP1*, *RAD51*, *MCM8*, *FEN1*). Another group of significantly up-regulated genes encode various histone proteins, *i*.*e*., *HIST2H3A*, *HIST1H2AB*, *HIST1H2BB*, *HIST1H2BM*, *HIST1H2AE*, which are essential for nucleosome core assembly.

**Table 2:** DAVID functional annotation clustering of upregulated genes in cEDS patients’ skin fibroblasts.

Cluster	Enrichment Score	Category	Term	FDR <0.3
1	9.2	GOTERM_BP	GO:0006260~DNA replication	2,24E^-08^
		GOTERM_BP	GO:0000082~G1/S transition of mitotic cell cycle	2,41E^-05^
2	3.5	UP_KEYWORDS	DNA damage	0,025
		GOTERM_BP	GO:0006281~DNA repair	1,288
3	3.2	INTERPRO	IPR001208: Mini-chromosome maintenance, DNA-dependent ATPase	0,131
		GOTERM_CC	GO:0042555~MCM complex	4,5774
		KEGG_PATHWAY	hsa04110: Cell cycle	4,730
4	2.4	GOTERM_CC	GO:0005654~nucleoplasm	2,244
5	2.3	UP_KEYWORDS	Cell cycle	0,001
		GOTERM_BP	GO:0051301~cell division	8,104
6	2.1	GOTERM_BP	GO:0000731~DNA synthesis involved in DNA repair	0,058
7	1.75	GOTERM_MF	GO:0004386~helicase activity	15,03
8	1.74	INTERPRO	IPR003593:AAA+ ATPase domain	18,96
9	1.2	INTERPRO	IPR002213: UDP-glucuronosyl/UDP-glucosyltransferase	27,2

The most enriched GO clusters of the 180 down-regulated DEGs (**Table 3** and **S4 Table**) were associated with genes related to the regulation of transcription (*ZNF630*, *CCDC85B*, *ZNF296*, *TRIB3*, *CCDC59*, *ZNF615*, *ZNF75D*, *HEY1*, *HAND2*, *XBP1*, *ZNF404*, *ZNF33A*, *EGR2*, *JUNB*, *ZNF415*). Many of these transcription-associated genes encode zinc finger domain-containing proteins that are involved in gene transcription and translation, mRNA trafficking, cytoskeleton organization, cell adhesion, protein folding, and chromatin remodeling. Different down-regulated genes, *i*.*e*., *CDKN1A*, *MDM2*, *PTEN*, *GADD45A*, *GADD45B*, are implicated in p53 and FOXO signaling pathways involved in the control of DNA replication, chromosome segregation and cell division.

**Table 3:** DAVID functional annotation clustering of down-regulated genes in cEDS patients’ skin fibroblasts.

Cluster	Enrichment Score	Category	Term	FDR <0.3
1	1.51	KEGG_PATHWAY	hsa04115: p53 signaling pathway	1,517
		KEGG_PATHWAY	hsa04068: FoxO signaling pathway	10,23
		GOTERM_BP	GO:0000079~regulation of cyclin-dependent protein serine/threonine kinase activity	22,22
2	1.43	GOTERM_BP	GO:0006355~regulation of transcription, DNA-templated	11,42
		INTERPRO	IPR013087: Zinc finger C2H2-type/integrase DNA-binding domain	24,27
		GOTERM_CC	GO:0005634~nucleus	23,40

Patients’ cells also showed decreased mRNA levels of several ECM-associated genes (**S2 Table**) including *SPP1*, *POSTN*, and *EDIL3*, which encode matricellular proteins primarily acting as mediators of cell–matrix interactions rather than as structural components. Specifically, osteopontin (*SPP1*) and the epidermal growth factor-like repeats discoidin I-like domain 3 protein (*EDIL3*), which contain an Arg-Gly-Asp (RGD) tripeptide integrin binding motif, and periostin (*POSTN*), containing an N-terminal EMI domain through which it binds to COLLI and FN, play an important role in cell proliferation and migration, cutaneous wound healing, cell cycle, and apoptosis. Likewise, *IGFBP2* and *C3* genes, showing up-regulated transcription in cEDS fibroblasts (**S2 Table**), encode insulin like growth factor binding protein 2 and complement C3, respectively, which are also involved in cell migration and wound repair process.

Finally, in cEDS fibroblasts biological functions related to intracellular protein transport, endoplasmic reticulum (ER) proteostasis, and autophagy seem to be perturbed, as suggested by the reduced transcription of many related genes such as *DNAJB7*, *RAB33A*, *VPS29*, *CCPG1*, *ATG10*, *SVIP*, and *VIPAS39* (**S2 Table**).

### Pathways enrichment analysis

To identify differentially expressed pathways in cEDS fibroblasts, an enrichment analysis was carried out on all DEGs by querying both ToppGene and DAVID databases and selecting a threshold of FDR-adjusted p-value <0.05. As shown in **S5 Table**, cell cycle was the most perturbed pathway in patients’ cells, as shown by a significant dysregulated expression of many cell cycle regulating genes. Some of these DEGs, *i*.*e*, *CDKN1A*, *MCM4*, *MCM5*, *MCM6*, *MCM10*, *RRM2*, *CDC45*, *TYMS*, *POLE2*, *MCM8*, *CCNE2*, and *CDC6*, are specifically involved in G1/S transition and G2/M checkpoint. DNA replication seems to be also induced, as indicated by the augmented transcription of different associated genes such as replication factor subunit 3 (*RFC3*), DNA polymerase epsilon 2 (*POLE2*), different members of the mini-chromosome maintenance complex (*MCM4*, *MCM5*, *MCM6*), and the flap endonuclease 1 (*FEN1*), all playing a crucial role in DNA replication and damage repair response.

Pathways analysis also indicated that p53 and FOXO signaling pathways seem to be perturbed in cEDS fibroblasts. These pathways are master regulators of cellular homeostasis in response to different stress signals including growth factor deprivation, metabolic stress, oxidative stress, and DNA mutations. Concerning the p53 signaling pathway, its perturbation is indicated by the dysregulated expression of several p53-regulated genes including cyclin E2 (*CCNE2*), cyclin-dependent kinase inhibitor 1A (*CDKN1A*), ribonucleotide reductase M2 (*RRM2*), MDM2 proto-oncogene (*MDM2*), phosphatase and tensin homolog (*PTEN*), and growth arrest and DNA-damage-inducible alpha (*GADD45A*) and beta (*GADD45B*). Regarding the FOXO signaling pathway, apart from *CDKN1A*, *MDM2*, *PTEN*, and *GADD45A*, cEDS cells also showed a higher expression of other FOXO related genes including polo-like kinase 1 (*PLK1*) and 4 (*PLK4*), SMAD family member 3 (SMAD3), and homer scaffold protein 2 (*HOMER2*).

Drug metabolism-related biological process was also disturbed, as indicated by the altered expression of glutathione S-transferase mu 3 (*GSTM3*), monoamine oxidase B (*MAOB*), and different members of UDP glucuronosyltransferase family 2, such as member B17 (*UGT2B17*), member A2 (*UGT2A2*), and member B7 (*UGT2B7*).

### Validation of microarray expression data by quantitative PCR

**Fig 2:**
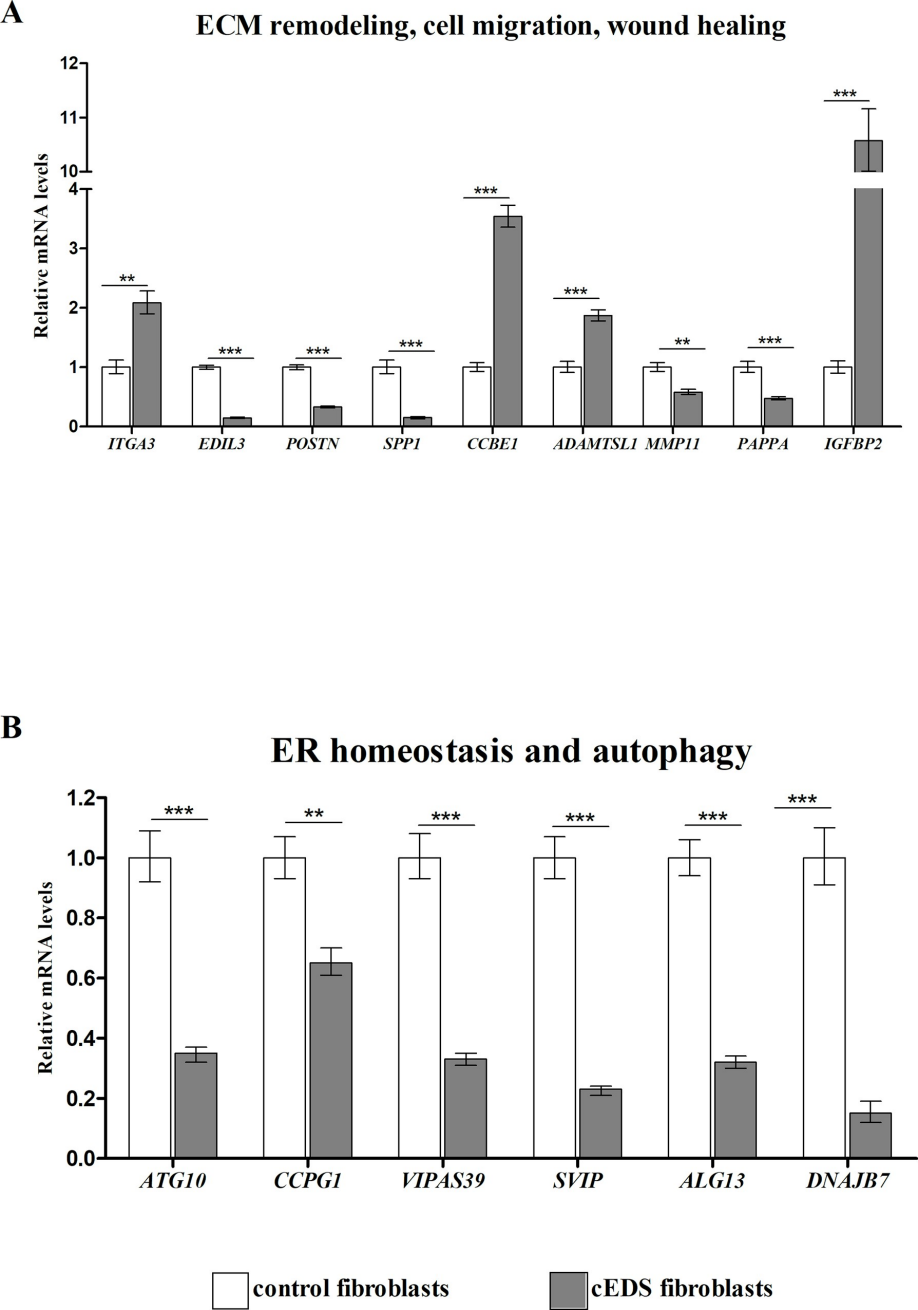
qPCR validation of genes related to ECM remodeling, wound healing, and ER homeostasis. (**A**) The relative mRNA expression levels of selected genes related to ECM architecture and (**B**) involved in ER homeostasis and autophagy, were determined with the 2^-(ΔΔCt)^ method normalized with the geometric mean of different reference genes. Bars represent the mean ratio of target gene expression in four patients’ fibroblasts compared to six unrelated healthy individuals. qPCR was performed in triplicate, and the results were expressed as mean ± SEM. **p<0.01, and ***p<0.001.

**Fig 3:**
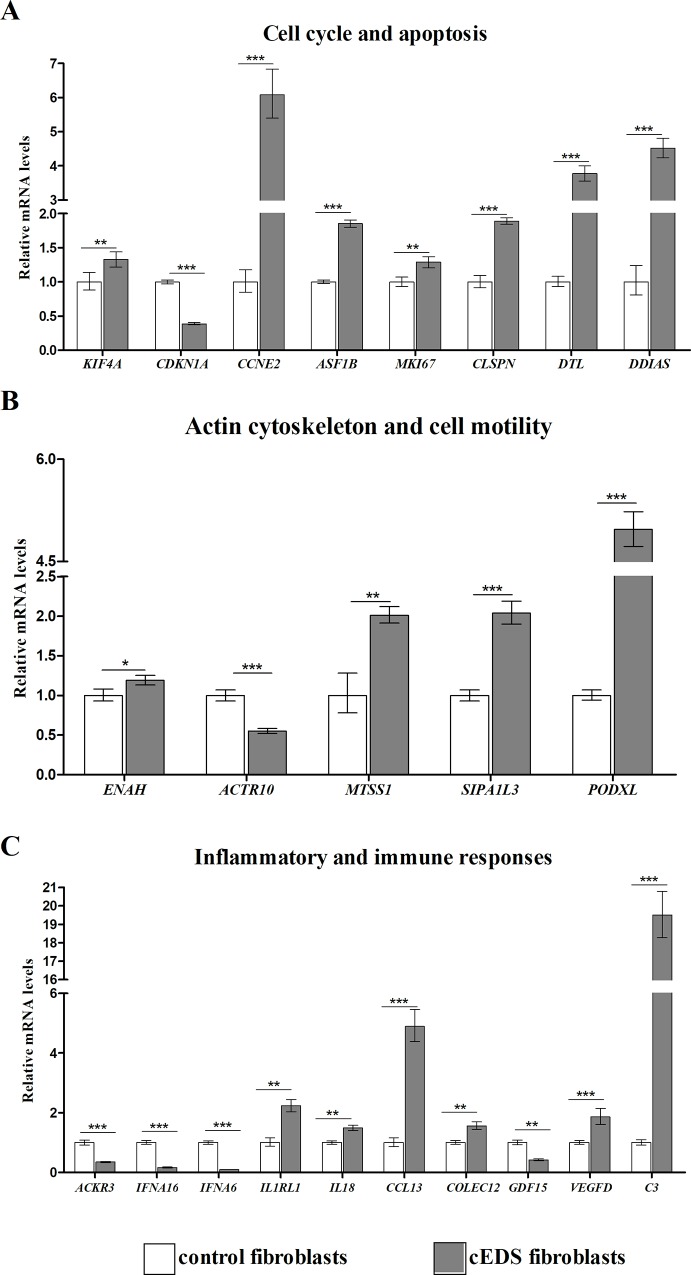
qPCR validation of genes involved in cell cycle regulation, actin cytoskeleton organization, and related to inflammatory and immune responses. **(A)** The relative mRNA expression levels of different cell cycle regulating genes, **(B)** transcripts associated with cytoskeleton organization, and **(C)** implicated in inflammation and immune response, were determined with the 2^-(ΔΔCt)^ method normalized with the geometric mean of different reference genes. Bars represent the mean ratio of target gene expression in four patients’ fibroblasts compared to six unrelated healthy individuals. qPCR was performed in triplicate, and the results were expressed as mean ± SEM. *p<0.05, **p<0.01, and ***p<0.001.

We evaluated the differential expression of a selection of DEGs by qPCR. Genes were prioritized based on their expression pattern and GO functional classification that was significantly perturbed in cEDS fibroblasts. Specifically, we focused on genes related to ECM remodeling, ER protein folding homeostasis/autophagy, inflammatory and immune response, cell cycle regulation, cell adhesion/motility and actin cytoskeleton organization (**Figs 2** and **3**). qPCR confirmed the differential expression of genes related to ECM remodeling, wound healing, and connective tissues integrity, such as *ITGA3*, *EDIL3*, *POSTN*, *SPP1*, *CCBE1*, *MMP11*, *PAPPA*, *ADAMTSL1*, and *IGFBP2* (**Fig 2A**). We also verified the altered expression of many transcripts related to ER homeostasis and autophagy, *i*.*e*., *ATG10*, *CCPG1*, *VIPAS39*, *SVIP*, *ALG13*, and *DNAJB7* (**Fig 2B**). Significant changes of mRNA levels of several cell cycle regulating genes. *i*.*e*., *KIF4A*, *CDKN1A*, *CCNE2*, *ASF1B*, *MKI67*, *CLSPN*, *DTL*, and *DDIAS*, were also confirmed (**Fig 3A**). qPCR also revealed altered transcriptional levels of different genes implicated in the cell adhesion and cytoskeleton organization, such as *ENAH*, *ACTR10*, *MTSS1*, *SIPA1L3*, and *PODXL* (**Fig 3B**), and involved in inflammatory and immune response, *i*.*e*., *ACKR3*, *IFNA16*, *IFNA6*, *CCL13*, *IL8*, *IL1RL1*, *COLEC12*, *VEGFD*, *GDF15*, and *C3* (**Fig 3C**).

### Aberrant COLLV expression causes the disassembly of different ECM structural proteins

**Fig 4:**
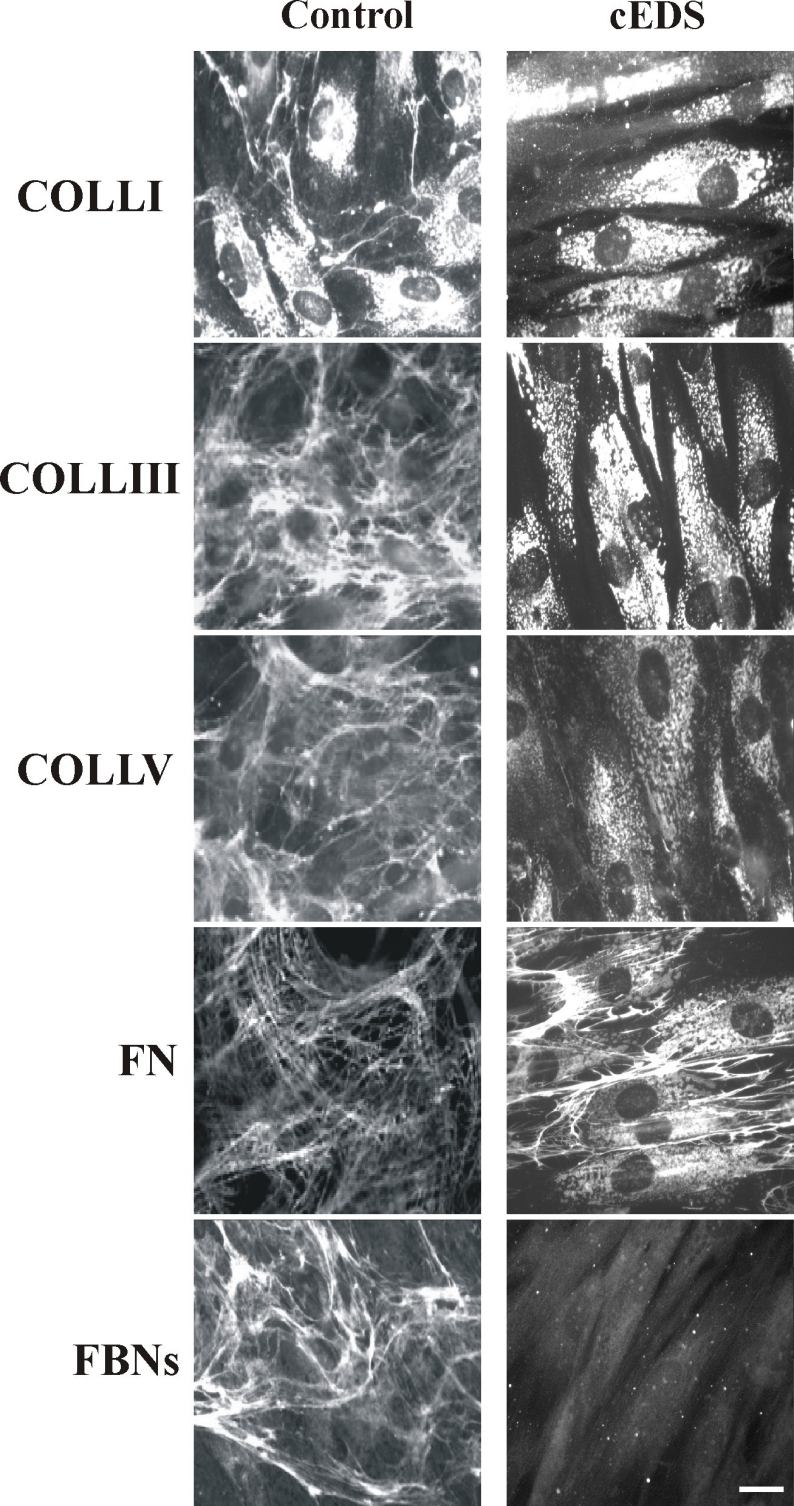
cEDS patients’ skin fibroblasts show the altered deposition of different structural components into the ECM. Control and patients’ cells were analyzed with specific Abs directed against COLLI, COLLIII, COLLV, FN, and FBNs. FN and FBNs were investigated by labeling the cells with Abs recognizing all their isoforms. Images are representative of 4 control and 4 cEDS cell strains. Scale bar: 10 μm.

In cEDS patients’ skin fibroblasts, we previously demonstrated abnormal synthesis and deposition of COLLV into the ECM together with the disarray of FN and the lack of COLLs- and FN-specific integrin receptors [15,16,20]. Moreover, these cells also showed an *in vitro* reduced migratory capability and poor wound healing rescued by purified exogenous COLLV treatment [14]. IF analysis of COLLV in the present patients’ cells confirmed the aberrant COLLV-ECM deposition and its intracellular retention (**Fig 4)**. The consequence of the aberrant COLLV expression on the organization of other ECM structural components such as COLLI, COLLIII, FN, and FBNs was investigated by IF. As reported in **Fig 4**, COLLI was accumulated in the cytoplasm with few thin ECM-fibrils in control fibroblasts, whereas it was only detected in the cytoplasm in patients’ cells. COLLIII was assembled into the ECM by control cells, but not by cEDS fibroblasts, in which this protein was retained in the cytoplasm. FN and FBNs were organized in fibrillar and differently shaped networks covering control fibroblasts, whereas these proteins were not assembled into the ECM in patients’ cells. Specifically, in cEDS fibroblasts only few FN fibrils were localized in the intercellular spaces, whereas FBNs were present in rare and sparse cytoplasmic spots (**Fig 4**).

## Discussion

**Fig 5:**
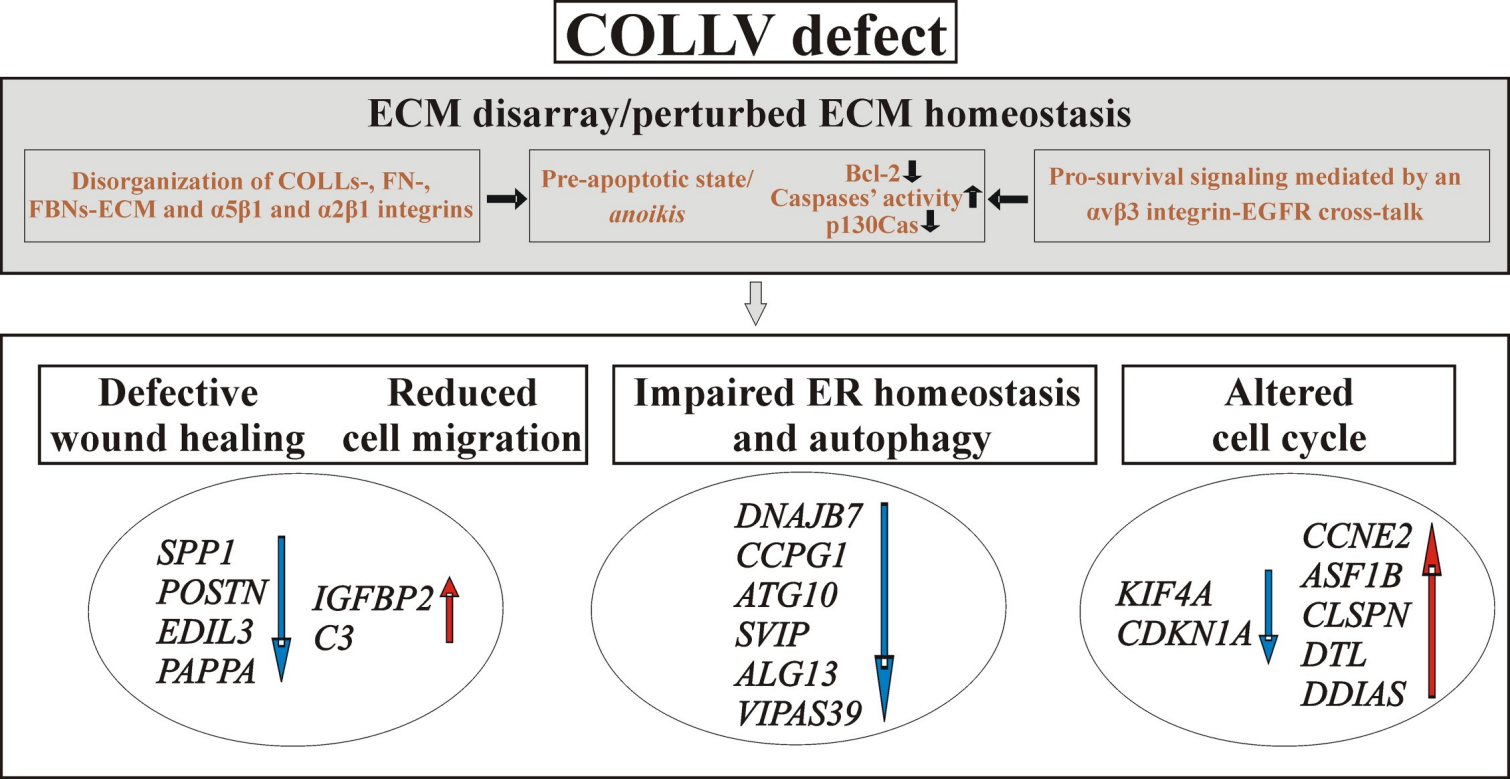
Schematic summary illustrating the main biological processes deregulated in skin fibroblasts with *COL5A1* and *COL5A2* pathogenetic variants. COLLV defect is associated with pathological ECM remodeling and *anoikis*, which is rescued by an αvβ3 integrin-EGFR signaling transduction pathway [20], together with defective ER homeostasis, autophagy and cell cycle progression. Down-regulated DEGs are indicated by blue arrows and those up-regulated by red arrows.

The present study reports the first molecular evidence of significant gene expression changes in dermal fibroblasts from cEDS patients. Although the reduction in the amount of COLLV is central to the pathogenesis of cEDS [26,27], so far, the molecular mechanisms contributing to the pathophysiology of the disease have never been investigated in-depth. Even if we recognize the limited number of analyzed samples and that findings should be confirmed in a larger cohort of patients, our results provide intriguing insights into dysregulated gene expression pattern and related biological processes that likely contribute to the molecular pathology of cEDS. We specifically focused on different candidate genes mainly involved in ECM remodeling, wound healing/inflammation, ER homeostasis/autophagy, and associated with cell cycle regulation (**Fig 5**).

### ECM remodeling, wound healing, and inflammation

Transcriptome profiling revealed the differential expression of a subset of genes, such as *SPP1*, *POSTN*, *EDIL3*, *IGFBP2*, *PAPPA*, *CCBE1*, *ITGA3*, *MMP11*, and *ADAMTSL1*, which are involved in ECM turnover, cell migration, and wound healing. After cutaneous injury, the wound healing occurs through several processes, including coagulation, granulation tissue formation, re-epithelialization, and ECM remodeling [28]. ECM components are essential for wound repair, since they create a provisional matrix, providing a structural integrity of matrix during healing process, and regulate the cell-cell and cell-matrix interactions [29,30]. ECM proteins like fibrin, FN, and COLLs contribute to the structural integrity of the matrix, whereas matricellular proteins, like osteopontin, periostin, tenascins, thrombospondins, and vitronectin, associate with the ECM and act temporally and spatially to provide signals eliciting specific cell activities within the wound [29,31]. Among the dysregulated ECM-related genes identified in cEDS fibroblasts, *SPP1* encodes osteopontin (OPN), a secreted phosphoprotein with an RGD motif through which it binds to cell surface receptors including αvβ3 integrin and CD44 [32]. OPN is particularly expressed during embryogenesis and participates in the resolution of dermal wounds [33]. Several *in vivo* and *in vitro* studies indicated that OPN promotes cell proliferation, cell migration, and wound healing through inhibition of apoptosis [33–36]. Interestingly, *Opn*-deficient mice show altered wound healing, with small COLL fibrils and disorganized ECM in the deep layers of the wound [33]. Despite these observations, a further work demonstrated by *in vivo* wound studies that the knockdown of OPN improves the skin wound healing and reduces scarring in mice [37]. Wu and colleagues [38] also demonstrated that OPN is a mediator of dermal fibrosis since stimulates TGFβ activation and increases dermal thickness in lesional skin of wild type compared to *Opn* deficient mice. In addition, dermal fibroblasts isolated from *Opn*^-/-^ mice show decreased migration compared to control cells, whereas treatment with OPN partially restores their migratory capability, suggesting an involvement of OPN in dermal fibroblasts migration [38].

*POSTN* encodes the matricellular protein periostin that is a predominantly expressed in COLLs-rich tissues and contributes to proper COLLs assembly and homeostasis [39,40]. It was previously reported that in bronchial tissues of patients with asthma periostin colocalizes with other ECM proteins such as COLLI, COLLIII, COLLV, FN, and tenascin-C [41], whereas COLLs cross-linking is significantly reduced in periostin-deficient mouse skin, suggesting a role of this ECM-associated matricellular protein in the appropriate COLLs fibrillogenesis [42].

Like other ECM proteins, periostin plays a fundamental role in tissue remodeling. Indeed, periostin interacts with αvβ3 and αvβ5 integrins on cell surfaces, influencing cell–matrix interactions, adhesion, proliferation, and differentiation [43]. In addition, periostin is a key modulator of processes essential for proper wound resolution [44]. In this regard, Ontsuka et al., [45] showed that periostin-deficient mice exhibit delayed *in vivo* wound repair capability that was improved by exogenous administration of periostin, indicating a role of this protein in the resolution of cutaneous wound repair. The authors also demonstrated that loss of periostin impairs proliferation and migration of healthy dermal fibroblasts that is rescued by exogenous supplementation of the protein, supporting its key role in the response to wounding. Moreover, loss of periostin affects COLLs stability and assembly in cultured human dermal fibroblasts, leading to higher amounts of COLLs fragments owing to proteases digestion [40]. Given the physiological role of periostin in the interaction with several ECM proteins and proper COLLs assembly to regulate the ECM architecture, its shortage may contribute to the generalized ECM disarray of cEDS patients’ fibroblasts [15,20, this study]. In this view, we previously demonstrated that cEDS fibroblasts exhibit a reduced migratory potential and delayed wound healing [14] owing to COLLV disorganization, defective fibrillar FN assembly into the ECM and abnormal integrin pattern [15,16,20,46], consistently with the poor wound repair and defective scars observed in cEDS patients [4]. Our expression and protein findings are also in line with *in vivo* wound healing studies on a *Col5a1*^*+/-*^ haploinsufficient murine model showing that these mutant mice heal significantly slower than the wild type [17], as in mice heterozygous for *Col5a2* null alleles that show deficits in wound healing [18]. In addition, dermal fibroblasts from *Col5a1*^*+/-*^ mice exhibit reduced proliferation, decreased attachment of fibroblasts to wound matrix components, and impaired cell migration [17].

*EDIL3*, which is the most down-regulated transcript in cEDS cells, encodes a matrix-associated matricellular protein with three epidermal growth factor (EGF)-like repeats, two discoidin-like domains, and an RGD motif in the second EGF-like repeat [47]. EDIL3 can induce angiogenesis through binding αvβ3 and αvβ5 integrins and increase neovascularization of ischemic tissues in a mouse model of hind-limb ischemia [48]. In endothelial cells, this ECM-related protein acts as a survival factor protecting these cells from apoptosis and *anoikis* through binding the RGD motif of the αvβ3 integrin [49]. In addition, its silencing impairs proliferative and migratory capabilities of human retinal endothelial cells *in vitro*, EGF receptor-signaling mediated pathways, and induces cell cycle arrest at the G1 phase [50]. Moreover, *Edil3*-deficient mice develop more severe osteoarthritis compared to control mice that is associated with increased susceptibility of chondrocytes to apoptosis [51].

The protein encoded by *IGFBP2*, which is most up-regulated transcript in cEDS fibroblasts, is a member of six similar proteins that bind insulin-like growth factors I and II (IGFs) modulating their activity. Other IGF-independent actions ascribed to IGFBP2 involve the C-terminal RGD motif, which mediates the interaction with the α5β1 integrin [52]. This interaction promotes cell migration in different cell models and stimulates the migratory capability of human dermal fibroblasts [52–56]. These IGFBP2-mediated functions suggest a possible involvement of this soluble protein in a potential rescue of migratory defects that was reported in cEDS fibroblasts and restored by the treatment with exogenous COLLV [14].

Furthermore, during tissue repair, an excessive inflammation delays healing and may lead to complications and chronic wounds [57]. Complement system activation is needed to restore tissue injury, however, its inappropriate activation causes cell death and enhances inflammation, thus contributing to further injury and impaired wound healing [58]. About this, in cEDS patients’ cells, we observed the dysregulated expression of many inflammatory and immune response related genes, *i*.*e*., *CCL13*, *IL8*, *IL1RL1*, *COLEC12*, *GDF15*, and *C3*. Of these, *C3*, showing an about 20-fold mRNA levels increase in patients’ fibroblasts, encodes a member of the complement system, which is composed of several plasma proteins interacting with numerous immune mediators involved in inflammatory response in several biological processes including wound healing [58]. Complement cascade can also regulate the migration and activation of immune cells such as macrophages and neutrophils, which are actively involved in wound healing [59]. Specifically, C3 seems to have an inhibitory role in wound healing [60]. Indeed, in *C3*^*-/-*^ mice C3 deficiency accelerates the initial rate of excisional wound healing, whereas wound healing is attenuated with sera from wild type animals or purified human C3 treatment. C3 deficiency also modulates the inflammatory infiltrate of healing by reducing the numbers of neutrophils and increasing the number of mast cells, thus accelerating the whole wound healing process [60]. Additionally, the pro-inflammatory effect of C3 exacerbates the skin inflammation in psoriasiform dermatitis [61].

Taken together, these gene expression changes suggest a potential involvement of different ECM-related proteins such as osteopontin, periostin, IGFBP2, and C3 in both the reduced migratory capability and wound repair of cEDS patients’ fibroblasts. More research is needed to establish the concrete role of these proteins in the process of cutaneous wound healing in cEDS cells.

The ADAMTS (a disintegrin-like and metalloproteinase with thrombospondin type 1 motifs) superfamily contains 19 secreted metalloproteinases involved in various biological processes including extracellular matrix (ECM) degradation, matrix assembly, angiogenesis, and cell migration.

### ER turnover, protein homeostasis, and autophagy

ER is a highly dynamic organelle in eukaryotic cells, which is deputed to lipid and protein biosynthesis, calcium storage, and detoxification of various exogenous and endogenous harmful compounds. Signaling sensors within the ER detect lumen perturbations and employ downstream cascades that engage effector mechanisms to restore homeostasis [62]. The most studied ER signaling mechanism is the unfolded protein response, which is known to increase many effector mechanisms, including autophagy [63]. This cellular defense mechanism is considered an effector pathway for ER stress through the process of autophagic sequestration of ER fragments into autophagosomes, namely ER-phagy. During ER-phagy a discrete portion of the ER is sequestered by nascent autophagosomes and delivered to lysosomes for degradation and recycling [64]. In this regard, our transcriptome profiling in cEDS cells revealed a decreased expression of different genes related to the ER protein folding homeostasis and autophagy, such as *DNAJB7*, *CCPG1*, *ATG10*, *SVIP*, and *VIPAS39*. Among them, the protein encoded by the *DNAJB7* belongs to the DnaJ heat shock protein family, which are molecular co-chaperones acting as ER reductases that catalyze the removal of non-native disulfides and enhance protein folding [65]. Cell-cycle progression gene 1 (*CCPG1*) encodes an ER-resident transmembrane protein that acts as a non-canonical autophagy cargo receptor essential for ER-phagy and ER proteostasis by binding Atg8-family proteins, thus preventing the hyperaccumulation of insoluble protein within the ER lumen [66]. The protein encoded by the autophagy related gene (*ATG10*) is an E2-like conjugating enzyme involved in two ubiquitin-like modifications essential for autophagosome formation, a double-membrane vesicle containing cellular debris that are degraded *via* lysosome-autophagy pathway [67]. *SVIP*, encoding the small p97/VCP-interacting protein, acts as an inhibitor of the ER-associated degradation pathway (ERAD) by competing with the E3 ligase binding to p97/VCP to regulate its function [68]. The p97/VCP protein is a member of the AAA ATPase family that plays a central role in ERAD, since it binds and extracts polyubiquitined protein from the ER for degradation by the cytosolic proteasomes [69]. The negative regulatory role of SVIP in ERAD leads to vacuoles formation that may be caused by accumulation of misfolded proteins when SVIP is overexpressed [70]. On the other hand, RNAi-mediated knockdown of *SVIP* reduces the levels of the LC3 microtubule-associated protein, which is involved in the autophagic pathway [71]. *VIPAS39* encodes a core constituent of the multiprotein tethering complexes, namely HOPS and CORVET complexes, which are essential to coordinate endosome and lysosome fusion in eukaryotic cells [72]. Specifically, VIPAS39 and the VPS33B partner protein control a myriad of functions including post-Golgi COLLs processing by regulation of the lysyl hydroxylase 3 (LH3) trafficking. LH3 is a member of the lysyl hydroxylase isoenzymes that catalyze procollagen lysine-hydroxylation, a post-translational modification essential for COLLs homeostasis [73]. Indeed, VIPAS39 and VPS33B deficiencies, which cause arthrogryposis, renal dysfunction and cholestasis syndrome (ARC), result in a reduction of LH3-dependent post-translational modification of COLLIV in inner medullary collecting duct cells accompanied by an abnormal ECM deposition [73]. Furthermore, cultured dermal fibroblasts from ARC patients show consistent procollagen I accumulation and structural COLLI defects were also found in tail tendons from *Vps33b* and *Vipas39* deficient mice [73]. Similarly, primary murine fibroblasts isolated from both mutant mice secrete a COLLI disorganized matrix, strengthening the evidence that defects in LH3 delivery cause alterations in COLLs modifications and cross-linking [74].

Both physiological and pathological changes in the composition of the ECM have been shown to alter autophagy activity. It is well known that many ECM components including COLLVI, decorin, and laminin α2 modulate autophagy both positively and negatively [75]. For instance, mice lacking COLLVI or decorin have impaired autophagic responses *in vivo* [76,77]. Besides, dermal fibroblasts critically depend on integrin-mediated cell adhesion to ECM for proper growth, proliferation, and survival, and the lack of cell adhesion to the ECM induces the fibroblasts’ growth arrest and *anoikis* [78]. Reduced ECM or integrin-mediated signals in primary human epithelial cells and mouse embryonic fibroblasts can induce autophagic flux, whereas autophagy regulators depletion enhances apoptosis in detached cells, confirming that induction of autophagy during *anoikis* promotes cell survival, thus acting as a persistence strategy to mitigate the stresses of ECM detachment [79]. In line with these observations, we previously demonstrated that the αvβ3 integrin-mediated signaling rescues from *anoikis* cEDS fibroblasts by a cross-talk with EGFR [20]. These findings are consistent with several evidences of cross-talk mechanisms between integrins and growth factors’ receptors eliciting cell growth and rescue from apoptosis [80,81]. Additionally, misfolded procollagen molecules containing mutations affecting triple helix conformation are likely to be degraded by the autophagy pathway, thus playing a protective role against ER stress [82]. Moreover, the treatment of Osteogenesis imperfecta patients’ skin fibroblasts with the autophagic inducer 4-phenylbutyric acid ameliorates autophagy, stimulates protein secretion and rescues cellular homeostasis, suggesting that a complementary process of autophagy may favor the degradation of retained unfolded proteins [83]. Conversely, the inhibition of autophagy and lysosome function in chondrocytes decreases COLLs transcription, as a possible compensatory mechanism to reduce the folding rate and to contrast accumulation of COLLs intermediates within the ER [64]. Taken together, all these evidences and our findings highlight that an appropriate autophagy is crucial to maintain ER homeostasis and COLLs proteostasis. Anyway, further investigations are required to verify the cellular consequences of a defective autophagy response in cEDS patients’ fibroblasts.

### Cell cycle

The prominent group of DEGs in cEDS cells was implicated in processes related to cell division, DNA replication, DNA repair, telomere organization, and associated with nucleosome and chromatin assembly. We also found significant expression changes of several transcripts encoding histone proteins that could reflect a perturbation of DNA synthesis, given that histones enter in nucleosome formation and proper DNA wrapping after the S phase of the cell cycle [84]. Besides, in cEDS fibroblasts, different up-regulated transcripts encode cell cycle regulators required for the G1/S transition (*CCNE2*, *KIF4A*, *DTL*), S phase progression (*MKI67*), and S/G2 transition (*CLSPN*), as well as components of the DNA replication machinery (*CDC6*, *CDC45*, *CDCA2*, *CDCA5*, *CDCA7*), which ensure sequential progression through the cell cycle in an orderly fashion [85]. Conversely, these cells show the diminished expression of the cyclin dependent kinase inhibitor 1A (*CKDN1A*), which encodes the p21 protein that is an inhibitor of cyclin-dependent kinase/cyclin complexes acting as a regulator of cell cycle progression at G1 [85]. Overall, the differential expression of several cell cycle related genes could suggest an attempt to overcome the G1 phase in response to the ECM deficiency-mediated pre-apoptotic state of these cells, in which caspases’ activity is up-regulated according to the low levels of the Bcl-2 anti-apoptotic protein [20]. In line with this hypothesis, cEDS fibroblasts also show increased mRNA levels of the DNA damage-induced apoptosis suppressor (*DDIAS*), which has an anti-apoptotic function during DNA damage by regulating caspase-8 stability and promoting its proteasomal degradation [86].

In conclusion, our approach illustrates global mRNA profiling changes of several genes and related biological processes that could offer novel insights in the cEDS pathophysiology. Additional functional work is required to verify whether altered ER proteostasis and defective autophagy play a crucial role in the pathogenesis of this connective tissue disorder, thus offering future therapeutic options.

## Supporting information

S1 Table Clinical and molecular findings of the cEDS patients. (DOCX)

S2 Table Full list of DEGs identified in cEDS patients’ skin fibroblasts. (XLSX)

S3 Table DAVID functional annotation clustering of 368 up-regulated DEGs in cEDS patients’ skin fibroblasts. (XLSX)

S4 Table DAVID functional annotation clustering of 180 down-regulated DEGs in cEDS patients’ skin fibroblasts. (XLSX)

S5 Table Top canonical pathways perturbed in cEDS patients’ skin fibroblasts. (XLSX)

## References

[1] Malfait F, Francomano C, Byers P, Belmont J, Berglund B, Black J, et al The 2017 international classification of the Ehlers-Danlos syndromes. Am J Med Genet C Semin Med Genet. 2017; 175: 8–26. 10.1002/ajmg.c.31552 28306229

[2] Bowen JM, Sobey GJ, Burrows NP, Colombi M, Lavallée ME, Malfait F, et al Ehlers-Danlos syndrome, classical type. Am J Med Genet C Semin Med Genet. 2017; 175: 27–39. 10.1002/ajmg.c.31548 28192633

[3] Colombi M, Dordoni C, Venturini M, Ciaccio C, Morlino S, Chiarelli N, et al Spectrum of mucocutaneous, ocular and facial features and delineation of novel presentations in 62 classical Ehlers-Danlos syndrome patients. Clin Genet. 2017; 92: 624–631. 10.1111/cge.13052 28485813

[4] Symoens S, Syx D, Malfait F, Callewaert B, Vanakker O, Coucke P, et al Comprehensive molecular analysis demonstrates type V collagen mutations in over 90% patients with classical EDS and allows to refine diagnostic criteria. Hum Mutat. 2012; 33: 1485–1493. 10.1002/humu.22137 22696272

[5] Ritelli M, Dordoni C, Venturini M, Chiarelli N, Quinzani S, Traversa M, et al Clinical and molecular characterization of 40 patients with classic Ehlers–Danlos syndrome: Identification of 18 COL5A1 and 2 COL5A2 novel mutations. Orphanet J Rare Dis. 2013; 8:58 10.1186/1750-1172-8-58 23587214 PMC3653713

[6] Colombi M, Dordoni C, Venturini M, Zanca A, Calzavara-Pinton P, Ritelli M. Delineation of Ehlers-Danlos syndrome phenotype due to the c.934C>T, p.(Arg312Cys) mutation in COL1A1: Report on a three-generation family without cardiovascular events, and literature review. Am J Med Genet A. 2017; 173: 524–530. 10.1002/ajmg.a.38035 28102596

[7] Schwarze U, Atkinson M, Hoffman G, Greenspan DS, Byers P. Null Alleles of the COL5A1 Gene of Type V Collagen are a cause of the classical forms of Ehlers–Danlos syndrome (types I and II). Am J Hum Genet. 2000; 66: 1757–1765. 10.1086/302933 10796876 PMC1378060

[8] Wenstrup RJ, Florer JB, Willing MC, Giunta C, Steinmann B, Young F, et al COL5A1 haploinsufficiency is a common molecular mechanism underlying the classical form of EDS. Am J Hum Genet. 2000; 66: 1766–1776. 10.1086/302930 10777716 PMC1378044

[9] Mak KM, Png CY, Lee DJ. Type V Collagen in Health, Disease, and Fibrosis. Anat Rec (Hoboken). 2016; 299: 613–29. 26910848 10.1002/ar.23330

[10] Birk DE. Type V collagen: Heterotypic type I/V collagen interacts in the regulation of fibril assembly. Micron. 1991; 32: 223–237. 10.1016/s0968-4328(00)00043-3 11006503

[11] Wenstrup RJ, Florer JB, Eric W, Brunskill EW, Bell SM, Chervoneva I, et al Type V collagen controls the initiation of collagen fibril assembly. J Biol Chem. 2004; 279: 53331–53337. 10.1074/jbc.M409622200 15383546

[12] Niyibizi C, Kavalkovich K, Yamaji T, Woo SL. Type V collagen is increased during rabbit medial collateral ligament healing. Knee Surg Sports Traumatol Arthrosc. 2000; 8: 281–285. 10.1007/s001670000134 11061296

[13] Shimomura T, Jia F, Niyibizi C, Woo SL. Antisense oligonucleotides reduce synthesis of procollagen alpha1 (V) chain in human patellar tendon fibroblasts: potential application in healing ligaments and tendons. Connect Tissue Res. 2003; 44: 167–172. 10.1080/03008200390215872 14504037

[14] Viglio S, Zoppi N, Sangalli A, Gallanti A, Barlati S, Mottes M, et al Rescue of migratory defects of Ehlers-Danlos syndrome fibroblasts in vitro by type V collagen but not insulin-like binding protein-1. J Invest Dermatol. 2008; 128: 1915–1919. 10.1038/jid.2008.33 18305566

[15] Zoppi N, Gardella R, De Paepe A, Barlati S, Colombi M. Human fibroblasts with mutations in COL5A1 and COL3A1 genes do not organize collagens and fibronectin in the extracellular matrix, down-regulate alpha2beta1 integrin, and recruit alphavbeta3 instead of alpha5beta1 integrin. J Biol Chem. 2004; 279: 18157–18168. 10.1074/jbc.M312609200 14970208

[16] Zoppi N, Chiarelli N, Binetti S, Ritelli M, Colombi M. Dermal fibroblast-to-myofibroblast transition sustained by αvß3 integrin-ILK-Snail1/Slug signaling is a common feature for hypermobile Ehlers-Danlos syndrome and hypermobility spectrum disorders. Biochim Biophys Acta. 2018; 1864: 1010–1023. 10.1016/j.bbadis.2018.01.005 29309923

[17] DeNigris J, Yao Q, Birk EK, Birk DE. Altered dermal fibroblast behavior in a collagen V haploinsufficient murine model of classic Ehlers-Danlos syndrome. Connect Tissue Res. 2016; 57: 1–9. 10.3109/03008207.2015.1081901 26713685 PMC4849881

[18] Park AC, Phan N, Massoudi D, Liu Z, Kernien JF, Adams SM, et al Deficits in Col5a2 Expression Result in Novel Skin and Adipose Abnormalities and Predisposition to Aortic Aneurysms and Dissections. Am J Pathol. 2017; 187: 2300–2311. 10.1016/j.ajpath.2017.06.006 28734943 PMC5809516

[19] Beighton P, De Paepe A, Steinmann B, Tsipouras P, Wenstrup RJ. Ehlers-Danlos syndromes: revised nosology, Villefranche, 1997. Ehlers-Danlos National Foundation (USA) and Ehlers-Danlos Support Group (UK). Am J Med Genet. 1998; 77: 31–37. 9557891 10.1002/(sici)1096-8628(19980428)77:1<31::aid-ajmg8>3.0.co;2-o

[20] Zoppi N, Barlati S, Colombi M. FAK-independent alphavbeta3 integrin-EGFR complexes rescue from anoikis matrix-defective fibroblasts. Biochim Biophys Acta. 2008; 1783: 1177–1188. 10.1016/j.bbamcr.2008.03.003 18405669

[21] Zoppi N, Chiarelli N, Cinquina V, Ritelli M, Colombi M. GLUT10 deficiency leads to oxidative stress and non-canonical αvβ3 integrin-mediated TGFβ signalling associated with extracellular matrix disarray in arterial tortuosity syndrome skin fibroblasts. Hum Mol Genet. 2015; 24: 6769–6787. 10.1093/hmg/ddv382 26376865 PMC4634379

[22] Chiarelli N, Carini G, Zoppi N, Dordoni C, Ritelli M, Venturini M, et al Transcriptome-Wide Expression Profiling in Skin Fibroblasts of Patients with Joint Hypermobility Syndrome/Ehlers-Danlos Syndrome Hypermobility Type. PLoS One. 2016; 11: e0161347 10.1371/journal.pone.0161347 27518164 PMC4982685

[23] Chiarelli N, Carini G, Zoppi N, Ritelli M, Colombi M. Transcriptome analysis of skin fibroblasts with dominant negative COL3A1 mutations provides molecular insights into the etiopathology of vascular Ehlers-Danlos syndrome. PLoS One. 2018; 13: e0191220 10.1371/journal.pone.0191220 29346445 PMC5773204

[24] Ritelli M, Chiarelli N, Zoppi N, Dordoni C, Quinzani S, Traversa M, et al Insights in the etiopathology of galactosyltransferase II (GalT-II) deficiency from transcriptome-wide expression profiling of skin fibroblasts of two sisters with compound heterozygosity for two novel B3GALT6 mutations. Mol Genet Metab Rep. 2014; 2: 1–15. 10.1016/j.ymgmr.2014.11.005 28649518 PMC5471164

[25] Benjamini Y, Hochberg Y. Controlling the false discovery rate: a practical and powerful approach to multiple testing. J R Stat Soc B Stat Methodol. 1995; 57: 289–300.

[26] Symoens S, Malfait F, Renard M, Andre J, Hausser I, Loeys B, et al COL5A1 signal peptide mutations interfere with protein secretion and cause classic Ehlers–Danlos Syndrome. Hum Mutat. 2008; 30: E395–E403. 10.1002/humu.20887 18972565

[27] De Paepe A, Malfait F. The Ehlers–Danlos syndrome, a disorder with many faces. Clin Genet. 2012; 82: 1–11. 10.1111/j.1399-0004.2012.01858.x 22353005

[28] Xue M, Jackson CJ. Extracellular matrix reorganization during wound healing and its impact on abnormal scarring. Adv Wound Care (New Rochelle). 2015; 4: 119–136. 25785236 10.1089/wound.2013.0485 PMC4352699

[29] Midwood KS, Williams LV, Schwarzbauer JE. Tissue repair and the dynamics of the extracellular matrix. Int J Biochem Cell Biol. 2004; 36: 1031–1037. 10.1016/j.biocel.2003.12.003 15094118

[30] Olczyk P, Mencner Ł, Komosinska-Vassev K. The role of the extracellular matrix components in cutaneous wound healing. Biomed Res Int. 2014; 2014: 747584 10.1155/2014/747584 24772435 PMC3977088

[31] Bornstein P, Sage EH. Matricellular proteins: extracellular modulators of cell function. Curr Opin Cell Biol. 2002; 14: 608–616. 12231357 10.1016/s0955-0674(02)00361-7

[32] Ailane S, Long P, Jenner P, Rose S: Expression of integrin and cd44 receptors recognising osteopontin in the normal and lps-lesioned rat substantia nigra. Eur J Neurosci 2013; 38: 2468–2476. 10.1111/ejn.12231 23692556

[33] Liaw L, Birk DE, Ballas CB, Whitsitt JS, Davidson JM, Hogan BL. Altered wound healing in mice lacking a functional osteopontin gene (spp1). J Clin Invest. 1998; 101: 1468–1478. 10.1172/JCI1122 PMC508725 9525990

[34] Takahashi F, Takahashi K, Okazaki T, Maeda K, Ienaga H, Maeda M, et al Role of osteopontin in the pathogenesis of bleomycin-induced pulmonary fibrosis. Am J Respir Cell Mol Biol. 2001; 24: 264–271. 10.1165/ajrcmb.24.3.4293 11245625

[35] Pardo A, Gibson K, Cisneros J, Richards TJ, Yang Y, Becerril C, et al Up-regulation and profibrotic role of osteopontin in human idiopathic pulmonary fibrosis. PLoS Med. 2005; 2: e251 10.1371/journal.pmed.0020251 16128620 PMC1198037

[36] Zhang H, Guo M, Chen JH, Wang Z, Du XF, Liu PX, et al Osteopontin knockdown inhibits αv,β3 integrin-induced cell migration and invasion and promotes apoptosis of breast cancer cells by inducing autophagy and inactivating the PI3K/Akt/mTOR pathway. Cell Physiol Biochem. 2014; 33: 991–1002. 10.1159/000358670 24714122

[37] Mori R, Shaw TJ, Martin P. Molecular mechanisms linking wound inflammation and fibrosis: knockdown of osteopontin leads to rapid repair and reduced scarring. J Exp Med. 2008; 205: 43–51. 10.1084/jem.20071412 18180311 PMC2234383

[38] Wu M, Schneider DJ, Mayes MD, Assassi S, Arnett FC, Tan FK, et al Osteopontin in systemic sclerosis and its role in dermal fibrosis. J Invest Dermatol. 2012; 132: 1605–1614. 10.1038/jid.2012.32 22402440 PMC3365548

[39] Hamilton DW. Functional role of periostin in development and wound repair: implications for connective tissue disease. J Cell Commun Signal. 2008; 2: 9–17. 10.1007/s12079-008-0023-5 18642132 PMC2570010

[40] Egbert M, Ruetze M, Sattler M, Wenck H, Gallinat S, Lucius R, et al The matricellular protein periostin contributes to proper collagen function and is downregulated during skin aging. J Dermatol Sci. 2014; 73: 40–48. 10.1016/j.jdermsci.2013.08.010 24055232

[41] Takayama G, Arima K, Kanaji T, Toda S, Tanaka H, Shoji S, et al, Periostin: a novel component of subepithelial fibrosis of bronchial asthma downstream of IL-4 and IL-13 signals. J Allergy Clin Immunol. 2006; 118: 98–104. 10.1016/j.jaci.2006.02.046 16815144

[42] Norris RA, Damon B, Mironov V, Kasyanov V, Ramamurthi A, Moreno-Rodriguez R, et al Periostin regulates collagen fibrillogenesis and the biomechanical properties of connective tissues. J Cell Biochem. 2007; 101: 695–711. 10.1002/jcb.21224 17226767 PMC3393091

[43] Kudo A. Periostin in fibrillogenesis for tissue regeneration: periostin actions inside and outside the cell. Cell Mol Life Sci. 2011; 68: 3201–3207. 10.1007/s00018-011-0784-5 21833583 PMC3173633

[44] Walker JT, McLeod K, Kim S, Conway SJ, Hamilton DW. Periostin as a multifunctional modulator of the wound healing response. Cell Tissue Res. 2016; 365: 453–465. 10.1007/s00441-016-2426-6 27234502 PMC5559884

[45] Ontsuka K, Kotobuki Y, Shiraishi H, Serada S, Ohta S, Tanemura A, et al Periostin, a matricellular protein, accelerates cutaneous wound repair by activating dermal fibroblasts. Exp Dermatol. 2012; 21: 331–336. 10.1111/j.1600-0625.2012.01454.x 22509828

[46] Zoppi N, Ritelli M, Colombi M. Type III and V collagens modulate the expression and assembly of EDA(+) fibronectin in the extracellular matrix of defective Ehlers-Danlos syndrome fibroblasts. Biochim Biophys Acta. 2012; 1820: 1576–1587. 10.1016/j.bbagen.2012.06.004 22705941

[47] Hidai C, Zupancic T, Penta K, Mikhail A, Kawana M, Quertermous EE, et al Cloning and characterization of developmental endothelial locus-1: an embryonic endothelial cell protein that binds the alphavbeta3 integrin receptor. Genes Dev. 1998; 12: 21–33. 9420328 10.1101/gad.12.1.21 PMC529342

[48] Zhong J, Eliceiri B, Stupack D, Penta K, Sakamoto G, Quertermous T, et al Neovascularization of ischemic tissues by gene delivery of the extracellular matrix protein Del-1. J Clin Invest. 2003; 112: 30–41. 10.1172/JCI17034 12840057 PMC162283

[49] Wang Z, Kundu RK, Longaker MT, Quertermous T, Yang GP. The angiogenic factor Del1 prevents apoptosis of endothelial cells through integrin binding. Surgery. 2012; 151: 296–305. 10.1016/j.surg.2011.07.013 21893328

[50] Shen W, Zhu S, Qin H, Zhong M, Wu J, Zhang R, et al EDIL3 knockdown inhibits retinal angiogenesis through the induction of cell cycle arrest in vitro. Mol Med Rep. 2017; 16: 4054–4060. 10.3892/mmr.2017.7122 28765888 PMC5646987

[51] Wang Z, Tran MC, Bhatia NJ, Hsing AW, Chen C, LaRussa MF, et al Del1 Knockout Mice Developed More Severe Osteoarthritis Associated with Increased Susceptibility of Chondrocytes to Apoptosis. PLoS One. 2016; 11: e0160684 10.1371/journal.pone.0160684 27505251 PMC4978450

[52] Mendes KN, Wang GK, Fuller GN, Zhang W. JNK mediates insulin-like growth factor binding protein 2/integrin alpha5-dependent glioma cell migration. Int J Oncol. 2010; 37: 143–153. 20514406 10.3892/ijo_00000662

[53] Schutt BS, Langkamp M, Rauschnabel U, Ranke MB, Elmlinger MW. Integrin-mediated action of insulin-like growth factor binding protein-2 in tumor cells. J Mol Endocrinol. 2004; 32: 859–868. 15171717 10.1677/jme.0.0320859

[54] Russo VC, Schütt BS, Andaloro E, Ymer SI, Hoeflich A, Ranke MB, et al Insulin-like growth factor binding protein-2 binding to extracellular matrix plays a critical role in neuroblastoma cell proliferation, migration, and invasion. Endocrinology. 2005; 146: 4445–4455. 10.1210/en.2005-0467 15994346

[55] Wang GK, Hu L, Fuller GN, Zhang W. An interaction between insulin-like growth factor-binding protein 2 (IGFBP2) and integrin alpha5 is essential for IGFBP2-induced cell mobility. J Biol Chem. 2006; 281: 14085–14091. 10.1074/jbc.M513686200 16569642

[56] Brandt K, Grünler J, Brismar K, Wang J. Effects of IGFBP-1 and IGFBP-2 and their fragments on migration and IGF-induced proliferation of human dermal fibroblasts. Growth Horm IGF Res. 2015; 25: 34–40. 10.1016/j.ghir.2014.11.001 25468444

[57] Wilgus TA. Immune cells in the healing skin wound: influential players at each stage of repair. Pharmacol Res. 2008; 58: 112–116 10.1016/j.phrs.2008.07.009 18723091

[58] Cazander G, Jukema GN, Nibbering PH. Complement activation and inhibition in wound healing. Clin Dev Immunol. 2012; 2012: 534291 10.1155/2012/534291 23346185 PMC3546472

[59] Ricklin D, Hajishengallis G, Yang K, Lambris JD. Complement: a key system for immune surveillance and homeostasis. Nat Immunol. 2010; 11: 785–797. 10.1038/ni.1923 20720586 PMC2924908

[60] Rafail S, Kourtzelis I, Foukas PG, Markiewski MM, DeAngelis RA, Guariento M, et al Complement deficiency promotes cutaneous wound healing in mice. J Immunol. 2015 194: 1285–1291. 10.4049/jimmunol.1402354 25548229 PMC4297721

[61] Giacomassi C, Buang N, Ling GS, Crawford G, Cook HT, Scott D, et al Complement C3 Exacerbates Imiquimod-Induced Skin Inflammation and Psoriasiform Dermatitis. J Invest Dermatol. 2017; 137: 760–763. 10.1016/j.jid.2016.11.011 27876407 PMC5319416

[62] Hegde RS, Ploegh HL. Quality and quantity control at the endoplasmic reticulum. Curr Opin Cell Biol. 2010; 22: 437–446. 10.1016/j.ceb.2010.05.005 20570125 PMC2929805

[63] Anelli T, Sitia R. Protein quality control in the early secretory pathway. EMBO J. 2008; 27: 315–327. 10.1038/sj.emboj.7601974 18216874 PMC2234347

[64] Settembre C, Cinque L, Bartolomeo R, Di Malta C, De Leonibus C, Forrester A. Defective collagen proteostasis and matrix formation in the pathogenesis of lysosomal storage disorders. Matrix Biol. 2018; 71–72: 283–293. 10.1016/j.matbio.2018.06.001 29870768

[65] Melnyk A, Rieger H, Zimmermann R. Co-chaperones of the mammalian endoplasmic reticulum. Subcell Biochem. 2015; 78: 179–200. 10.1007/978-3-319-11731-7_9 25487022

[66] Smith MD, Harley ME, Kemp AJ, Wills J, Lee M, Arends M, et al CCPG1 is a non-canonical autophagy cargo receptor essential for ER-phagy and pancreatic ER proteostasis. Dev Cell. 2018; 44: 217–232. 10.1016/j.devcel.2017.11.024 29290589 PMC5791736

[67] Lock R, Debnath J. Extracellular matrix regulation of autophagy. Curr Opin Cell Biol. 2008; 20: 583–588. 10.1016/j.ceb.2008.05.002 18573652 PMC2613490

[68] Ballar P, Zhong Y, Nagahama M, Tagaya M, Shen Y, Fang S. Identification of SVIP as an endogenous inhibitor of endoplasmic reticulum-associated degradation. J Biol Chem. 2007; 282: 33908–33914. 10.1074/jbc.M704446200 17872946

[69] Ballar P, Pabuccuoglu A, Kose FA. Different p97/VCP complexes function in retrotranslocation step of mammalian ER-associated degradation (ERAD). Int J Biochem Cell Biol. 2011; 43: 613–621. 10.1016/j.biocel.2010.12.021 21199683

[70] Ballar P, Fang S. Regulation of ER-associated degradation via p97/VCP-interacting motif. Biochem Soc Trans. 2008; 36: 818–822. 10.1042/BST0360818 18793143

[71] Wang Y, Ballar P, Zhong Y, Zhang X, Liu C, Zhang YJ, et al SVIP induces localization of p97/VCP to the plasma and lysosomal membranes and regulates autophagy. PLoS One. 2011; 6: e24478 10.1371/journal.pone.0024478 21909394 PMC3164199

[72] Balderhaar HJ, Ungermann C. CORVET and HOPS tethering complexes-coordinators of endosome and lysosome fusion. J Cell Sci. 2013; 126: 1307–1316. 10.1242/jcs.107805 23645161

[73] Banushi B, Forneris F, Straatman-Iwanowska A, Strange A, Lyne AM, Rogerson C, et al Regulation of post-Golgi LH3 trafficking is essential for collagen homeostasis. Nat Commun. 2016; 7: 12111 10.1038/ncomms12111 27435297 PMC4961739

[74] Rogerson C, Gissen P. VPS33B and VIPAR are essential for epidermal lamellar body biogenesis and function. Biochim Biophys Acta. 2018; 1864: 1609–1621. 10.1016/j.bbadis.2018.01.028 PMC5906731 29409756

[75] Neill T, Schaefer L, Iozzo RV. Instructive roles of extracellular matrix on autophagy. Am J Pathol. 2014; 184: 2146–2153. 10.1016/j.ajpath.2014.05.010 24976620 PMC4116694

[76] Grumati P, Coletto L, Sabatelli P, Cescon M, Angelin A, Bertaggia E, et al Autophagy is defective in collagen VI muscular dystrophies, and its reactivation rescues myofiber degeneration. Nat Med. 2010; 16: 1313–1320. 10.1038/nm.2247 21037586

[77] Gubbiotti MA, Neill T, Frey H, Schaefer L, Iozzo RV. Decorin is an autophagy-inducible proteoglycan and is required for proper in vivo autophagy. Matrix Biol. 2015; 48: 14–25. 10.1016/j.matbio.2015.09.001 26344480 PMC4661125

[78] Frisch SM, Francis H. Disruption of epithelial cell-matrix interactions induces apoptosis. J Cell Biol. 1994; 124: 619–626. 8106557 10.1083/jcb.124.4.619 PMC2119917

[79] Fung C, Lock R, Gao S, Salas E, Debnath J. Induction of autophagy during extracellular matrix detachment promotes cell survival. Mol Biol Cell. 2008; 19: 797–806. 10.1091/mbc.E07-10-1092 18094039 PMC2262959

[80] Yamada KM, Even-Ram S. Integrin regulation of growth factor receptors. Nat Cell Biol. 2002; 4: E75–76. 10.1038/ncb0402-e75 11944037

[81] Reginato MJ, Mills KR, Paulus JK, Lynch DK, Sgroi DC, Debnath J, et al Integrins and EGFR coordinately regulate the pro-apoptotic protein Bim to prevent anoikis. Nat Cell Biol. 2003; 5: 733–740. 10.1038/ncb1026 12844146

[82] Ishida Y, Yamamoto A, Kitamura A, Lamande SR, Yoshimori Y, Bateman JF, et al Autophagic elimination of misfolded procollagen aggregates in the endoplasmic reticulum as a means of cell protection. Mol Cell Biol. 2009; 20: 2744–2754. 10.1091/mbc.E08-11-1092 PMC2688553 19357194

[83] Besio R, Iula G, Garibaldi N, Cipolla L, Sabbioneda S, Biggiogera M, et al 4-PBA ameliorates cellular homeostasis in fibroblasts from osteogenesis imperfecta patients by enhancing autophagy and stimulating protein secretion. Biochim Biophys Acta. 2018; 1864: 1642–1652. 10.1016/j.bbadis.2018.02.002 PMC5908783 29432813

[84] Zentner GE, Henikoff S. Regulation of nucleosome dynamics by histone modifications. Nat Struct Mol Biol. 2013; 20: 259–266. 10.1038/nsmb.2470 23463310

[85] Lim S, Kaldis P. Cdks, cyclins and CKIs: roles beyond cell cycle regulation. Development. 2013; 140: 3079–3093. 10.1242/dev.091744 23861057

[86] Im JY, Kim BK, Lee JY, Park SH, Ban HS, Jung KE, et al DDIAS suppresses TRAIL-mediated apoptosis by inhibiting DISC formation and destabilizing caspase-8 in cancer cells. Oncogene. 2018; 37: 1251–1262. 10.1038/s41388-017-0025-y 29242605

