# Supplementary material for: Molecular insights in the pathogenesis of classical Ehlers-Danlos syndrome from transcriptome-wide expression profiling of patients’ skin fibroblasts
Source: PLoS One. 2019 Feb 4;14(2):e0211647. doi: 10.1371/journal.pone.0211647 (PMC6361458; doi:10.1371/journal.pone.0211647)
Supplement: S1 Table — (DOCX) [file pone.0211647.s001.docx]

**S1 Table. Clinical and molecular findings of the cEDS patients.**

| **Patients** | **P1**  AN_002514  according to [4] | **P2**  AN_002503  according to [4] | **P3**  AN_002526 according to [4] | **P4**  AN_002534  according to [4] |
| --- | --- | --- | --- | --- |
| **Gender** | F | M | M | F |
| **Age (y)** | 34 y | 40 y | 46 y | 39 y |
| **Gene/Mutation** | *COL5A1*  c.2988del  (p.Gly997Alafs*77) | *COL5A1*  c.1165-2A>G  (p. Pro389Leufs*168) | *COL5A1*  c.4178G>A  (p. Gly1393Asp) | *COL5A2*  c.2499+2T>C  (p. Gly816_Pro833del) |
| **Functional consequence on COLLV** | Haploinsufficiency  (positive null-allele test) | Haploinsufficiency  (positive null-allele test) | Dominant negative effect | Dominant negative effect |
| ***Major criteria according to the 2017 EDS nosology [1]*** | | | | |
| **Skin hyperextensibility and atrophic scars** | + | + | + | + |
| **Generalized joint hypermobility (Beighton score)** | + (5/9) | + (9/9) | - (4/9) | + (9/9) |
| ***Minor criteria*** | | | | |
| **Easy bruising** | + | + | + | + |
| **Soft, doughy skin** | + | + | + | + |
| **Skin fragility** | + | + | + | + |
| **Molluscoid pseudotumors** | - | + | - | + |
| **Subcutaneous spheroids** | - | - | + | - |
| **Hernia (or a history thereof)** | - | - | - | + |
| **Complications of joint hypermobility** (e.g., sprains, luxation/subluxation, pain, flexible flatfoot) | +  Subdislocations  Chronic articular pain | +  Pes planus | +  Recurrent dislocations  Chronic/generalized pain | +  Recurrent dislocations  Chronic articular pain  Vertebral dislocations (surgically treated)  Pes planus |
| **Family history of a first degree relative who meets clinical criteria** | - | + | - | - |
| **Others** | Follicular hyperkeratosis  Plantar creases  Scoliosis  Sacroiliitis  Right pulmonary artery  hypoplasia | Piezogenic  papules  Varicose veins | Piezogenic papules  Blue sclerae  High palate Microretrognathia  Onychodystrophy Scoliosis Spondylolisthesis  Bilateral hallux valgus  Pes cavus  Arthrosis Tendinopathy Carpal tunnel  syndrome  Chronic fatigue syndrome | Piezogenic papules  Anisocoria  Congenital kyphoscoliosis  (surgically treated)  Bilateral valgus  knees  Hallux valgus  Hip periarthritis Arthrosis  Tendinopathy  Enthesopathies  Osteoporosis  Muscle hematomas  and rupture  Mitral valve prolapse  Chronic fatigue syndrome  Urethral and  rectal prolapse |

Abbreviations: F, female; M, male; +, present, -, absent; y, years. DNA mutation numbering is based on the cDNA sequence, and the +1 position corresponds to the A of the ATG translation initiation codon in the reference sequence. (*COL5A1*: NM_000093.3, NP_000084.3, *COL5A2*: NM_000393.31, NP_000384.2).
